# Self-Perception of Quality of Life and Emotional Well-Being among Students Attending Hospital Classrooms during COVID-19 Pandemic

**DOI:** 10.3390/healthcare9080943

**Published:** 2021-07-26

**Authors:** Laia Riera-Negre, Berta Paz-Lourido, Francisca Negre, María Rosa Rosselló, Sebastià Verger

**Affiliations:** 1 GREID Research Group, University of the Balearic Islands, 07122 Palma, Spain; laia.rierainegre@gmail.com; 2 Hospital Pedagogy Lab, Department of Nursing and Physiotherapy, Research Institute of Health Sciences (IUNICS), Institute of Research and Innovation in Education (IRIE), University of the Balearic Islands, Cra. De Valldemossa km 7.5, 07122 Palma, Spain; 3 Hospital Pedagogy Lab, Department of Applied Pedagogy and Psychology of Education, Institute of Research and Innovation in Education (IRIE), University of the Balearic Islands, 07122 Palma, Spain; xisca.negre@uib.es (F.N.); mrosa.rossello@uib.es (M.R.R.); s.verger@uib.es (S.V.)

**Keywords:** COVID-19, quality of life, emotional well-being, education, hospital classroom, pandemic

## Abstract

The COVID-19 pandemic caused disruptions in schooling and the closure of schools worldwide, how this has affected children’s and youth’s health and wellbeing is a current area of research. However, those who suffer a chronic or temporary disease may be attending hospital classrooms, and this scenario has received little attention in comparison to regular schools. The objective of this exploratory quantitative study focuses on exploring the quality of life and emotional well-being of students attending hospital classrooms. For this purpose, four Chilean hospital classrooms from different regions of the country were randomly selected. A total number of 248 students participated in the survey, each of whom filled out two online questionnaires. The findings show similar scores in children with mental illness and those with other health conditions. In comparison with one year before, students rate their general health as the same or somewhat better now, as well as manifesting an optimistic view of the future regarding the pandemic.

## 1. Introduction

The health crisis derived from COVID-19 has affected the entire society and a large number of services including education institutions that were forced to adopt measures to prevent and contain the spread of the virus [1]. According to UNESCO [2], at the beginning of 2020, schools interrupted their classes in more than 190 countries, affecting 1.57 billion children and young people, representing 90% of the world’s student population. These closures were carried out as a quick anti-virus measure, but in many countries the school closures ended up remaining almost indefinite, caused by the lack of health safety to reopen schools [3].

Education administrations and schools are entitled to address the consequences of these disruptions in schooling and ensure the health and safety of students, their families, and school personnel [4]. Furthermore, although the scale, extent, and duration of the COVID-19 crisis makes it difficult to draw uniform conclusions about its effects on young children, some children will undoubtedly need additional support to overcome the negative impacts on their health and emotional development [5,6,7,8,9]. It is relevant to take into consideration that school is not only an education resource but, as a social environment that impacts social wellbeing, in many places it also ensures the access to benefits that families cannot provide to their own children [10,11,12,13]. Although the adaptation of education in times of the COVID-19 pandemic has been different depending on regional educational system and resources, the virtualization of teaching has been a frequent but challenging response in many countries, with the child population attending school from their homes [14,15].

When children are sick or face chronic diseases, education can be developed in different ways [16], but the education in hospital classrooms is an alternative to ensure the right for children’s education [17,18,19]. The so-called hospital pedagogy is a discipline that integrates educational and psychoeducational actions aimed at people suffering from various diseases and their families, trying to respond to their biopsychosocial needs, to develop their potentialities, and improve their quality of life [20,21,22].

In the case of Chile, the Ministry of Education has developed a wide-ranging framework to ensure that education reaches all the students who, due to health reasons, cannot attend normalized schooling, either temporarily or permanently [23]. There are currently 56 hospital classrooms dependent on the Ministry of Education spread throughout the different Chilean regions. These hospital classrooms are comprised of different education professionals and provide educational support tailored to the needs of each student within their context, whether it be in the hospital or convalescing at home. It is, therefore, crucial to know the impact that this change of course on educational proposals has had on the schooling, quality of life, and emotional well-being of children with chronic diseases in Chile. To address this, a series of activities are designed and implemented with the participation of professionals in the area of health and education, giving sense to multidisciplinary work between teams [24]. The role of teachers who work in hospital classrooms is not alien to challenges, since they must bear in mind the various situations or conditions of each student, whether they are receiving classes at the hospital or from their home [25].

Given this scenario, knowing the perception that children and young people have about their quality of life and their emotional well-being may help to obtain a general exploration of the pandemic’s impact on their health and wellbeing. As part of a broader multi-center research project, the aim of this study is to identify the health and emotional wellbeing among students attending Chilean hospital classrooms.

## 2. Participants and Methods

### 2.1. Participants

Participants were students attending 4 hospital classrooms in different regions of Chile. The hospital classrooms were randomly selected from all those in the country.

### 2.2. Instruments

Quality of life and health perception were measured using the Short Form 36 Health Survey (SF-36), a survey designed by the Health Institute, New England Medical Center, Boston Massachusetts [26] and has been extensively validated in an American context [27,28,29]. The Spanish version, used in this study, has been validated and used in numerous studies for decades [30,31,32,33,34]. This instrument is a non-disease-specific short-form survey developed to assess multiple health indicators, including function, distress and well-being, and health self-evaluations. The questionnaire consists of 36 items measuring the following eight dimensions of life quality: Physical Functioning; Physical Role, which refers to role limitations due to physical difficulties; Bodily Pain; General Health; Vitality; Social Functioning; Emotional Role, which refers to role limitations due to emotional difficulties; and Mental Health. Response choices vary from two to six. There are many instruments to measure quality of life [34], but the SF-36 includes one single item regarding perceived differences in state of health over the past year. This item is particularly relevant in this study due to the fact that the COVID-19 pandemic has affected people worldwide during this past year and to date. The use of SF-36 is not recommended with young children, but it has been successfully used with young adolescents, teenagers, and young adults [35,36,37]. In this age group, online self-administration has not demonstrated differences with other ways of administration [38].

In addition to the evaluation of emotional and mental health through the SF-36, emotional wellbeing was also studied with another instrument. This was an adaptation from another previous questionnaire developed by Ojala [39], with the objective of analyzing the emotional well-being of children and young people in relation to climate change, since this topic is starting to be recognized as a stressor for young generations. The main aims of Ojala’s study were to explore how a group of Swedish 12-year-olds cope with global climate change and to examine how different coping strategies relate to well-being (life satisfaction, negative affect, positive affect) and environmental engagement (environmental efficacy, pro-environmental behavior), as well as optimism concerning climate change and a sense of purpose in life.

In current times, the COVID-19 pandemic could be considered as a stressor for students, such that how they cope with this threat could be important for both engagement and psychological well-being. This instrument was translated into Spanish, and the topic “Climate Change” was substituted with “COVID-19”. For example, the question “Please indicate on a scale of 1 to 6 how much you worry about the negative consequences caused by climate change” was changed to “Please indicate on a scale of 1 to 6 how much you worry about the negative consequences caused by the COVID-19 crisis”. This adapted instrument consisted of 26 questions.

### 2.3. Procedure and Analysis

This exploratory online survey was anonymous, and data obtained did not allow for the identification of participants, the hospital pedagogy program or region at any stage of the study. Furthermore, data were analyzed by external researchers with no contact or any direct involvement in the hospital pedagogy programs studied. Anonymity and informed consent were ensured using a two-question strategy that gauged participant understanding of the consent process [40]. Descriptive information was asked about year of birth, location (hospital classrooms/home), health-related condition in the generic dimensions of heart disease, rare disease, mental illness, and others, but no concrete diagnosis was asked for at any time. Parents, teachers, and participants were informed and agreed to participate in this study. Data were analyzed by independent researchers that did not meet the students, and all information was stored following the regulations and ethical issues in research as well as legal requirements for personal data protection [41]. Data analysis was developed with SPSS 19.

## 3. Results

A total number of 248 students answered the survey. Both the mean and the median age were 15 years old (See Table 1). In the case of the “mental illness” group, the average was 16 years of age; in contrast, “heart disease” had an average age of 9 years old. The rest of the groups revolved around 13 and 14 years old; moreover, the majority suffered from mental illness (60.49% of the total), followed by rare diseases (10.08% of the total).

### 3.1. Normality Test

The Normality test using the Shapiro–Wilk statistic shows a significance *p* > 0.05 in all the items in both factors, except for the mental illness group *p* (*MI*) *=* 0.001 (see Table 2).

### 3.2. Standarized Scores

The participants’ score is calculated by adding the scores of each item in each of the factors. These factors are quality of life (QoL) and emotional well-being (EWB). The maximum score indicates a high presence of these factors, while a low score indicates a low quality of life or emotional well-being. QoL ranges from a minimum score of 30 to a maximum of 169, while EWB ranges from 26 to 133. In order to interpret these results, the direct scores are transformed into standardized Z scores. The emotional well-being (EWB) variable’s mean is *m* = −0.0711 and the standard deviation *SD* = 1.0225. The lowest score is EWB (*min*) = 3.4685, well below the lower limit and the 25th percentile, while the maximum value is EWB (*max*) = 1.93813, indicating that the Gaussian bell is shifted to the right (See Table 3). The area of highest density on the normal distribution of the EWB variable is in the interval [−1.0936, 0.9514]. The quality of life (QoL) variable’s mean is *m* = −0.0396 and the standard deviation *SD* = 1.0162. The QoL (*min*) = −2.97702, which is also well below the mean, and the maximum value is QoL (*max*) = 2.1409; therefore, the Gaussian bell is also skewed to the right. The area of highest density in the Gaussian bell for the QoL variable is located in the interval [−1.0558, 0.9766]. The QoL factor reliability test presents a Cronbach’s alpha a (QoL) = 0.908 for a total of 33 items, which is above the fixed limit value and indicates that there is a high degree of internal consistency. However, the reliability statistic for the EWB factor is (EWB) *=* 0.713 for 26 items, which indicates an acceptable internal consistency.

### 3.3. Descriptive Statistics of the Standarized Scores

Focusing on the results obtained according to the disease suffered by the participants, it is observed that the EWB factor’s mean for patients with heart disease is the lowest, *m* (*HD*) = −1.2422, followed by mental diseases, *m* (*MI*) *=* −0.485, which are below the factor’s mean. On the other hand, participants with other types of illness have the highest EWB, *m* (*Other*) *=* 0.1417 (see Table 4). In the case of the QoL factor depending on the disease, the group of rare diseases *m* (*RD*) *=* −0.6614 and heart disease *m* (*HD*) *=* −0.3346 are below the factor’s mean, while the rest are found above. In this case, the participants in the mental illness group have the highest perceived QoL with *m* (*MI*) *=* 0.0753 (see Table 5). On the other hand, it is observed in Table 5 that the 25th and 50th Tukey’s percentiles hinges of the heart disease subcategory are much lower than those of the other categories, as well as the EWB factor’s mean. In the case of the QoL factor, the RD subcategory has all the percentiles shifted to the left, which is confirmed by comparing them with the mean.

### 3.4. Study Location (Home vs. Hospital Classroom)

Most of the participants attended classrooms physically located at hospitals (*n* = 232), while others followed the educational intervention from their homes (16). In relation to the difference in means depending on study location, the EWB factor in the participants who stay at home shows a mean of *m* = 0.003, while in the group that attends the hospital classroom it is *m* = −0.007. Regarding the QoL factor, the mean is *m* (home) = −0.115 versus *m* (hospital classroom) = 0.003 (see Table 6). To see if the differences in the means are statistically significant, the ANOVA test for independent samples is applied. Assuming equality of variances in both factors, in the case of EWB the significance *p* > 0.005 and the confidence interval contains 0; therefore, there is no difference between the two means. In a similar way, in the case of the QoL factor, the significance *p* = 0.651 (>0.05) is not statistically significant either (see Table 7).

### 3.5. QoL Factor

We wanted to analyze the subjects’ perception of the different factors in relation to the COVID-19 pandemic. In relation to the QoL factor, it is of interest to analyze the perception of the participants’ own health, current and prior to COVID-19. Item number one assesses their health at the time of answering the questionnaire, while item number two assesses their health one year before answering the questionnaire. In item number one, it is observed that, in all cases, regardless of the disease, that participants rate their health as fair (two) or good (three), as seen in Table 8. It is observed that the sum of the accumulated percentage of these two represents 76.53% of the total. However, in item number two, most of the participants state that their health is similar to that of a year ago (40.74%), followed by 21.39% who state that this year, during the pandemic, their health is “somewhat better” (see Table 9).

### 3.6. EWB Factor

Regarding the EWB, item three contains three questions that refer to the expectations of the participants regarding the resolution of the COVID-19 crisis. In the case of question one, “I am hopeful that the COVID-19 crisis will be resolved in the future”, most of the participants agree that “It applies a lot”, being 55.96% of the total. Question two refers to whether the participant believes that we will resolve the COVID-19 crisis in the future and, in the same way, most of the participants agree that we will (55.14% of the total). Finally, regarding “I think the future looks bright when it comes to the COVID-19 crisis”, the response percentages are more equitable (see Table 10).

### 3.7. Mental Illness vs. Non-Mental Illness

Non-mental illnesses were grouped into a single group because the sample of each of them is small compared to the group of mental illnesses, which has a significantly larger sample. This way we can analyze whether there are differences between mental illnesses against all the rest. The mean age in the “Non-mental illness” group is *m* = 13 years, in comparison to the “mental illness” group for which, as previously mentioned, it is *m* = 16 years, (see Table 11).

Regarding the difference in means according to the type of illness, due to the reduced number of samples for the types of illnesses that are not “mental illness”, they have been grouped into the same group called “Non-mental illness” (see Table 12); in this way, the sample of both groups is better compensated (146 vs. 94). In the case of the EWB factor, the significance *p* = −0.901 (>0.05), which indicates that there is no difference between the two means. On the other hand, in the case of QoL, a greater difference is observed between the means of both groups, although this mean is slightly higher than the limit value; therefore, it cannot be stated that the difference is statistically significant, *p* = 0.09 (>0.05). This is corroborated from the confidence interval that contains the value 0 (see Table 13).

## 4. Discussion

The assessment of QoL presents many coincidences with respect to the EWB, in the case of children attending hospital pedagogy programs. Despite the fact that the evaluations are very close to the average, we can see how for an important part or for the majority, value is given to the impact that these two factors really have on their own lives. On occasions when the valuation is higher than the average, in no case it is “very good” or “excellent”. The valuations revolve around the central values on the positive side of the distribution, being “average” and “good”, while the negative valuations are far from the average and are more extreme. From here, we can deduce that chronic diseases have symptoms that affect their lives in diverse ways and intensities, which has been widely studied in children and young people with different pathological conditions [42].

In order to seek the impacts of the pandemic in this population group, questions one and two of the QoL factor are particularly relevant. It is striking that, in general, participants scored this item as “about the same” or even “somewhat better than one year ago”, which indicates a better QoL now. This fact may seem contradictory at first, and gives us clues to think that, on the one hand, it might be conditions related to health care issues, such as a good response to any recent treatment, or even obtaining the needed health care assistance. However, on the other hand, it is necessary to understand that the pandemic crisis could somewhat normalize their situation compared to their peers at school, because many of the new situations (closures, online education, lack of social contact) were already part of the daily lives of those students with chronic diseases.

As previously mentioned, this study was carried out on a sample of 248 students. Of these, participants with mental illness represent approximately 60% of the sample. Although this represents a high percentage of the sample, it is necessary to take into account not only the prevalence of mental illnesses in the Chilean population [43,44,45], but also the increasing support and visibility that these people have, for example being included in hospital pedagogy programs, while in other countries or regions, these adolescents could be enrolled in regular schools while still receiving psychotherapeutic treatment. Taking into account the stigma or stereotypical view of mental illnesses and, therefore, the risk of bullying that young people may suffer in school settings, the option of hospital pedagogy may be an alternative for certain phases of the illness or children’s conditions. These programs might be giving support to students affected by diseases but added to that, might also protect them from the repercussions found in schoolchildren derived from the COVID-19 pandemic. In this sense, studies are already showing impacts at the level of anxiety, depressions, and other mental disorders and situations, such as social distancing, increased pressure on families, reduced access to support services, or exposure to violence, that have been aggravated by the pandemic [7,8,9].

Regarding the other identified diseases, oncological diseases and heart disease, these cannot be taken as representative, since the sample is very small. It is also significant that the children classified in the others category obtained higher scores. Possibly, this is due to the fact that the variety of diseases represented within this category of others, do not present such a serious symptomatology or are affected as negatively as the rest of the categories, that is, the impact on their quality of life and their emotional well-being is not perceived as that serious.

Relative to EWB, the results suggest that participants, despite their QoL not reaching certain levels, are still optimistic and have these positive expectations that this pandemic situation can be resolved in the future. It is important to have hopeful future expectations to score high in EWB. It is, therefore, important to continue studying the long-term impacts of COVID-19, not only in regular schools but also with children and young people who follow other schooling programs in order to identify the strategies that students have used to cope with this situation [46].

Finally, it is important to keep in mind several limitations of this study. First, due to its general and exploratory nature, specific sociodemographic features that could have helped to better understand the impacts of the pandemic in this particular group of the population were not considered. In addition, the existence of standardized instruments to assess health-related quality of life and wellbeing in minors and young adults adapted to different pathologies and contexts are well known, but in this case a non-specific disease questionnaire was used. The SF-36 was originally developed to identify different dimensions impacting QoL instead of one single QoL factor, but, in this study, SF-36 was used together with another questionnaire, an adaptation from another previous instrument not specifically developed for this purpose. A final limitation, as discussed above, is that there is no control group without disease; therefore, comparisons and analysis are intragroup. This means that the level of QoL and the EWB of the participants in relation to the rest of the population cannot be known, but a participant can be positioned in relation to the rest of the participants. This is due to the fact that because of the urgency of the situation, a control group could not be established. Several measures to reduce the response bias were taken into account, avoiding any data, logotype, or information that could be interpreted by participants as an answer preference for this research. Besides being anonymous, the standardized questionnaires included neutrally worded questions.

As a future implication of this study, there is a need to gain in depth understanding of the reasons why adolescents attending hospital pedagogy programs do not refer the expected impacts in their health and wellbeing, and this must be contrasted with information from the families and their own perception regarding the educational and health care received. That is, what are the strengths, in order to reinforce them and what are the weaknesses, in order to improve on them. To address all these issues, in a further study, an adapted questionnaire [47] might be used with which we would obtain this information; moreover, including a qualitative phase in a new study would also allow us to obtain in depth contextualized data with recommendations to follow, not only for possible future confinements, but to gain a better understanding of the impact COVID-19 has on children with chronic diseases and to think about future care strategies should the pandemic drag on.

## 5. Conclusions

Chronic diseases affect QoL and EWB, and these two factors are directly related. Added to that, the COVID-19 pandemic is causing different impacts in school children worldwide. However, data from this study do not show a very significant impact on the QoL for the sample studied, and the perception of their health status was equal or not worse than one year ago. We must not lose sight of the fact that much of the information that has already been obtained during the pandemic might be useful to those children who, due to their illness, are unable to attend school in person. However, while other children are already normalizing their educational situation in many places worldwide with the re-opening of schools, children with chronic illnesses involved in hospital pedagogy programs will cease to participate in the experience of a normalized educational situation where no child attended school in person, to belong to an exceptional space such as the hospital or home care. For this reason, the return to normality for many healthy children may be a return to a perception of exceptionality for sick children that must be studied, since the impacts that have not appeared in times of pandemic could appear at later stages of returning back to normality. These students will need to be monitored in order to obtain tailored responses to their specific needs.

## Figures and Tables

**Table 1:** Descriptive table according to type of disease.

Type of Disease	Frequency	Percentage	Accumulated Percentage	Year of Birth
Rare Disease	25	10.08%	10.08%	2007
Heart Disease	3	1.20%	11.28%	2012
Oncological Disease	9	3.62%	14.90%	2007
Mental Illness	150	60.49%	75.40%	2005
Other	61	24.60%	100%	2008
Total	248	100%		2006

**Table 2:** Normality test.

Factor	Items	Statistical	Degrees of Freedom	*p*
EWB	Rare Disease	0.854	5	0.208
	Heart Disease	0.964	24	0.515
	Oncological Disease	0.955	9	0.750
	Mental Illness	0.965	146	0.001
	Other	0.957	59	0.035
QoL	Rare Disease	0.986	5	0.962
	Heart Disease	0.960	24	0.434
	Oncological Disease	0.952	9	0.712
	Mental Illness	0.990	146	0.349
	Other	0.962	59	0.062

**Table 3:** Factors’ standardized scores.

	Mean	SD	Mean’s Standard Error	Minimum	Maximum	Percentile 25	Percentile 50	Percentile 75	Cronbach’s Alpha
Emotional well-being (EWB)	−0.0711	1.0225	0.0653	−3.4685	1.9381	−0.6459	0.1889	0.6659	0.908
Quality of life (QoL)	−0.0396	1.0162	0.0645	−2.97702	2.1409	−0.7518	0.0269	0.8058	0.713

**Table 4:** Descriptive statistics of the emotional well-being factor according to the disease variable.

Disease	*N*	Mean	SD	Median	Min	Max	P25	P50	P75
Rare Disease	25	0.0563	0.8015	0.1491	−1.2422	1.7791	−0.4471	0.1491	0.4671
Heart Disease	3	−1.2422	2.4737	−1.2422	−2.9914	0.5069	−2.9914	−1.2422	0.5069
Oncological D.	9	−0.0496	0.6310	0.0298	−0.9242	0.9045	−0.6061	0.2989	0.3479
Mental Illness	150	−0.485	1.0423	0.1491	−3.4685	1.9381	−0.6856	0.1491	0.6659
Other	61	0.1417	1.004	0.2684	−2.4391	1.6996	−0.4074	0.2684	0.8249

**Table 5:** Descriptive statistics of the quality-of-life factor according to the disease variable.

Disease	*N*	Mean	SD	Median	Min	Max	P25	P50	P75
Rare Disease	25	−0.6614	1.1909	−1.0021	−2.9770	1.6402	−1.2803	−1.0021	0.1938
Heart Disease	3	−0.3346	1.2980	−0.3346	−1.2525	0.5832	−1.2524	−0.3346	0.5832
Oncological D.	9	0.05171	0.7141	0.1382	−0.9743	1.0839	−0.3624	0.1382	0.6945
Mental Illness	150	0.0753	0.9389	0.02699	−2.6988	2.1409	−0.5849	0.0269	0.8058
Other	61	0.0524	1.0970	0.0269	−1.9756	2.1409	−0.6683	0.0269	1.0283

**Table 6:** Difference of means according to study location (home vs. hospital classroom).

	Study Location	*n*	Mean	SD
EWB	Home	16	0.003	1.277
	Hospital classroom	232	−0.007	1.007
QoL	Home	16	−0.115	0.931
	Hospital classroom	232	0.003	1.023

**Table 7:** Independent samples test.

Levene’s Equality of Variances Test							Equality of Means *t*-Test		
		F	*p*	t	df	*p* Bilateral	Means Difference	95% Lower Confidence Interval	95% Upper Confidence Interval
EWB	Equal variances are assumed	0.578	0.448	0.041	248	0.967	0.011	−0.526	0.549
	Equal variances are not assumed			0.033	15.157	0.974	0.011	−0.705	0.727
QoL	Equal variances are assumed	0.140	0.709	−0.453	248	0.651	−0.119	−0.637	0.398
	Equal variances are not assumed			−0.492	17.595	0.629	−0.119	−0.629	0.390

**Table 8:** Item number 1 of the QoL factor.

In General, You Would Say that Your Health Is...	RD	HD	O	MI	Other	%
Poor	2	1	0	7	1	3.3%
Fair	14	0	3	51	27	39.09%
Good	7	1	3	59	21	37.44%
Very good	2	0	0	17	4	9.46%
Excellent	0	0	3	13	7	9.46%

**Table 9:** Item number 2 of the QoL factor.

Compared to One Year Ago, How Would You Rate Your Health in General Now?	RD	HD	O	MI	Other	%
Much worse now	0	0	0	6	2	3.29%
Somewhat worse now	6	0	2	16	7	12.75%
About the same	10	1	3	61	24	40.74%
Somewhat better now	8	1	2	36	13	21.39%
Much better now	1	0	2	28	14	18.51%

**Table 10:** Item number 3 (questions 1 to 3) of the EWB factor.

		RD	HD	O	MI	Other	%
I hope that the COVID-19 crisis will be resolved in the future	Does not apply	0	0	0	12	1	5.34%
	2	2	0	0	5	3	4.11%
	3	3	1	1	33	9	19.34%
	4	3	0	3	24	4	13.99%
	Applies a lot	16	1	5	72	42	55.96%
I believe we will resolve the COVID-19 crisis in the future	Does not apply	2	0	0	12	2	6.58%
	2	2	0	0	6	3	4.52%
	3	1	1	1	32	8	17.69%
	4	4	0	1	26	5	14.81%
	Applies a lot	15	1	7	70	41	55.14%
I think the future looks bright when it comes to the COVID-19 crisis	Does not apply	6	0	2	33	12	21.81%
	2	3	0	1	16	6	10.69%
	3	2	1	2	40	15	24.69%
	4	4	1	0	29	7	16.87%
	Applies a lot	7	0	4	28	19	23.86%

**Table 11:** Age means according to the type of illness (mental health vs. non-mental health).

	Mean	
	Mental Illness	Non-Mental Illness
Year of birth	2005	2008

**Table 12:** Difference of means according to the type of illness (mental illness vs. non-mental illness).

	Type of Illness	*n*	Mean	SD
EWB	Mental illness	150	−0.048	1.042
	Non-mental illness	98	0.072	0.964
QoL	Mental illness	150	0.072	0.936
	Non-mental illness	98	−0.148	1.113

**Table 13:** Independent samples test.

Levene’s Equality of Variances Test							Equality of Means *t*-Test		
		F	*p*	t	df	*p* Bilateral	Means Difference	95% Lower Confidence Interval	95% Upper Confidence Interval
EWB	Equal variances are assumed	0.537	0.464	−0.901	248	0.368	−0.120	−0.384	0.143
	Equal variances are not assumed			−0.917	209.423	0.360	−0.120	−0.380	0.138
QoL	Equal variances are assumed	5.227	0.023	1.666	248	0.097	0.220	−0.040	0.481
	Equal variances are not assumed			1.607	178.222	0.110	0.220	−0.050	0.138

## Data Availability

The data presented in this study are available on request from the corresponding author.
